# Neuronal Ceroid Lipofuscinoses Type 7 (CLN7)- A Case Series Reporting Cross Sectional and Retrospective Clinical Data to Evaluate Validity of Standardized Tools to Assess Disease Progression, Quality of Life, and Adaptive Skills

**DOI:** 10.21203/rs.3.rs-3983366/v1

**Published:** 2024-06-26

**Authors:** Saima Kayani, Veronica Bordes Edgar, Andrea Lowden, Emily R Nettesheim, Hamza Dahshi, Souad Messahel, Berge A Minassian, Benjamin M Greenberg

**Affiliations:** UT Southwestern: The University of Texas Southwestern Medical Center; UT Southwestern: The University of Texas Southwestern Medical Center; UT Southwestern: The University of Texas Southwestern Medical Center; UT Southwestern: The University of Texas Southwestern Medical Center; UT Southwestern: The University of Texas Southwestern Medical Center; UT Southwestern: The University of Texas Southwestern Medical Center; UT Southwestern: The University of Texas Southwestern Medical Center; UT Southwestern: The University of Texas Southwestern Medical Center

**Keywords:** MFSD8, neuronal ceroid lipofuscinosis, Batten disease, rare brain disease

## Abstract

**Background:**

This study evaluated the clinical characteristics of neuronal ceroid lipofuscinosis type 7 or CLN7 disease spectrum to characterize the clinical, electrophysiologic and neuroimaging phenotypes.

**Methods:**

We performed a single-center cross sectional data collection along with retrospective medical chart review in patients with a genetic diagnosis of CLN7. This study received ethical approval by the University of Texas Southwestern Medical Center Institutional Review Board. A total of 8 patients were included between the ages of 4 to 6 years. All patients had a genetic diagnosis of CLN7 with homozygous or compound heterozygous mutations in the *MFSD8* gene. The information collected includes patient demographics, developmental history, neurological events including seizures and neurodevelopmental regression along with further evaluation of brain magnetic resonance imaging and electrophysiological findings. The clinical phenotype is described through cross sectional and retrospective data collection and standardized tools assessing quality of life and functional skills.

**Conclusions:**

Our findings in this cohort of CLN7 patients indicated that development is initially normal with onset of clinical symptoms as early as two years of age. Language problems were noted prior to or at the onset of seizures in all cases. Gait problems were noted prior to seizure onset in 3 of 8 patients, and at or within 6 months after the onset of seizures in 5 of 8 patients. All patients followed a progressive course of language, motor, and neurocognitive deterioration. Congruent with the medical history, our patients had significantly low scores on adaptive abilities. Natural history data such as this can be used to support future clinical trial designs.

## Introduction

Recent advances in gene therapy have enabled development of potentially disease-modifying therapies for monogenic neurodegenerative diseases like neuronal ceroid lipofuscinosis type 7 (CLN7).

CLN7 is an autosomal recessive disorder which is caused by homozygous or bi-allelic heterozygous variants in the CLN7/MFSD8 gene. This gene is typically inherited from healthy, carrier parents each contributing a defective copy. The *CLN7/MFSD8* gene encodes a 518-amino acid polytopic lysosomal transmembrane protein with 12 membrane-spanning domains^1,2^. After the initial identification of the *MFSD8* gene in 2007, a total of 67 different *MFSD8* mutations have been reported in populations throughout the world (Table 1). The types of mutations include missense, splice site, nonsense, frame shift, sequence deletion or insertion^3^.

The clinical spectrum can vary from a mild, late onset with non-syndromic visual deficits^4^ to a severe, early-onset version that can manifest with progressive deterioration in intellectual and motor capabilities, seizures, muscle spasms and visual deficits culminating in premature death.^5^

## Materials and Methods

### Study Design and population

Ethical approval was obtained from the University of Texas Southwestern Medical Center Institutional Review Board prior to commencing this research study (STU 2020-0916, STU 2020-0640). An informed consent waiver was obtained for all participants. We performed a single-center cross sectional data collection and a retrospective medical chart review that included 8 patients with genetic diagnosis of CLN7. The data was collected during a natural history study of CLN7 for four patients over the course of 1 year. Baseline assessments and retrospective clinical data is reported here. The remaining four patients are currently seen in our center as part of a gene therapy clinical trial. For the patients enrolled in the clinical trial, only baseline clinical assessments are reported along with retrospective data that was collected before the initial screening visit.

Patients were eligible for the study if they were under the age of 18 years and had a confirmed genetic diagnosis of CLN7, defined as two pathogenic variants in the *MFSD8* gene. Collected data included longitudinal sociodemographic characteristics, brain MRI (Magnetic Resonance Imaging) findings, clinical presentations, laboratory results, operative reports if available, and medications. Motor and cognitive development was also reviewed, and data was collected on motor functions and intellectual ability. *CLN7* mutation type, age of symptom onset, age of diagnosis, regression (motor, language, or cognitive), presenting neurologic signs and symptoms, and developmental milestones were collected. Electrophysiologic data was collected per clinical standards and EEG data was analyzed by a board-certified epileptologist.

Participants were assessed by a board-certified neuropsychologist who administered standardized measures with both the child and their caregivers. Individual assessments with the children were done in their native language either through the bicultural/bilingual neuropsychologist (certified in Spanish) or a professional interpreter when English or Spanish was not the native language. Professional interpreters received training instructions in how to assist in the evaluation. The following measures were administered in this order: Mullen Scales of Early Learning (Mullen)^6^, Vineland Adaptive Behavior Scales, 3^rd^ Edition Comprehensive Interview (Vineland-3)^7^, Infant Toddler Quality of Life Questionnaire (ITQOL)^8^ for children under 5 years and the Quality of Life Inventory-Disability (QI-Disability)^9,10^ for children 5 years of age and older. The natural history patients (participants 1–4) only received the Mullen and Vineland-3. Tests were selected to accurately assess each patient based on the child’s individual abilities and stamina even when used outside of the age range (i.e., Mullen).

The Mullen is a test of early cognitive ability and motor development. It is made up of five scales: Gross Motor, Fine Motor, Visual Reception (visual problem-solving), Receptive Language and Expressive Language. The latter four make up the Early Learning Composite^6^. The raw scores were converted to age equivalents and subsequently to developmental quotients (age equivalent/chronological age * 100) as opposed to norm-referenced scores. While this test has limitations in the age of data and test items, this test was selected over other measures due to its brevity and ease in administration with more impaired participants. The normative data, although dated, extends to 68 months which is further than other infant measures. Given the decline in participants, raw data and age equivalents are utilized to capture the decline or progress in development of participants. Given the low functioning of participants, standard scores are rarely used, therefore, Flynn effect and other concerns with using dated norms do not apply to measured change over time. The Mullen also allows the researchers to separate out expressive and receptive language abilities as well as motor functioning which may have differential impact based on the disease process. Further, vision loss in participants will contribute to performance across any standardized measure making the separation of language and motor functioning from problem-solving even more necessary.

Adaptive behavior can be defined as the performance of daily activities required for personal and social sufficiency. Adaptive behavior is impacted in virtually all disorders that cause cognitive/developmental regression. The Vineland-3^7^ was utilized to assess adaptive behavior. This test was administered in a structured interview format with the participant’s caregivers to reduce the impact of parental reporting bias on scores. Scores are norm-referenced standard scores for the domains (SS mean = 100, SD = 15) and v-scale scores for sub-domains (mean = 15, SD = 3). Further, developmental quotients were also calculated for language and motor scales.

Infant Toddler Quality of Life Questionnaire (ITQOL)^8^- Short form is a 47-item measure to assess physical and psychosocial functioning of children ages 2 months to 5 years. It contains 12 scales including physical functioning, growth and development, bodily pain, temperament and moods, general behavior, getting along, general health perceptions, parental impact: emotional, parental impact: time, family activities, family cohesion, and change in health. Scores transformed to scale of 0 to 100 with higher scores indicating better quality of life.

Quality of Life Inventory-Disability is a measure to capture the health and well-being of children ages 5–18 with intellectual disability (QI-Disability)^9,10^. This measure was particularly developed to assess children with a wide range of disabilities and has been validated on children with Rett Syndrome, Down Syndrome, and CDKL5 deficiency disorder. Both Rett Syndrome and CDKL5 deficiency disorder are most like our study population. The validation on children with CDLK5 deficiency disorder spanned children as young as 3 years of age. This 32-item measure assesses a child’s social interactions, positive emotions, physical health, negative emotions, leisure skills, and independence. Scores are a 5-point Likert scale and transformed to scale of 0 to 100 with higher scores indicating better quality of life.

Given this is an ultra-rare disease, we wanted to be inclusive of participants regardless of linguistic and cultural differences. This is reflected in the diverse geographic and cultural representation of participants. Cultural and linguistic considerations were made across measures including the use of interpreters. Performance-based measures (i.e., Mullen) included nonverbal or very brief instructions given the task items that were being administered. Parents also remained in the room as is appropriate with the Mullen and were able to validate the child’s performance as consistent with their current functioning. The adaptive measure (i.e., Vineland-3) is also scored based on clinician judgment from the interview questions asked. This reduces the reliance on the interpreter needing to interpret verbatim and allows for tailoring questions to the parents in a way they will understand. While the same interpreter was not always available for follow-up evaluations, the same examiner was used which allowed for consistency in the clinical judgment. Possible bias was reduced on the part of the examiner as scores from previous evaluations are not available to the examiner after they are submitted to the study team and medical records are not reviewed. See our other work for more detailed information in the cultural considerations^11^.

A 21-channel video-EEG recording was obtained and analyzed in each patient. Electrodes were placed using the 10–20 nomenclature system. Photic stimulation was performed from 1–20Hz with 10 second trials and testing with the eyes opened and closed. The recording was of at least 60 minutes in duration in all 8 patients.

### Data collection

Electronic medical records from UTSW/Children’s Health Dallas with a genetically confirmed diagnosis of CLN7 were reviewed. Deidentified data was abstracted into a password protected spreadsheet for analysis.

## Results

In the cohort under study, a total of eight patients were enrolled, with ages spanning from 4–6 years. The studies group participants consisted of 5 females and 3 males. All the patients included in this study were from distinct families and were unrelated to each other. The collected dataset is described below and encompasses the demographic information, genotypic profile, key clinical, neuroimaging and electrophysiologic characteristics. Among the clinical phenotypic spectrum, our group focused on studying the onset and progression of neurologic symptomatology and comprehensive neuropsychological evaluation.

### Demographics/Genotype:

All 8 patients were clinically diagnosed with CLN7 deficiency, and the diagnosis was molecularly confirmed with homozygous or biallelic heterozygous pathogenic mutations in *MFSD8* gene. The genetic diagnosis was made through epilepsy gene panels, whole exome sequencing and *MFSD8* sequence analysis. Descriptive analysis was used to analyze the data which revealed heterogeneity of geographic, cultural, and linguistic and demographics backgrounds as detailed in Table 2.

### Clinical Characteristics:

#### Birth history, early development, and age of onset of symptoms:

All patients (8 out of 8) were reported to have an uncomplicated birth and an unremarkable early post-natal period. Early development was normal until 2 years of age.

The earliest symptom onset was at two years of age. In all the patients (8 out of 8) the onset of symptoms was noted before 4 years of age (Table 3) and the age of diagnosis was between 4 and 5 years. In our study cohort, median age of onset of symptoms is 3.4 years and median age of diagnosis is 4.8 years.

In the study cohort, the initial presenting symptoms prompting the families to seek medical attention varied among the patients. Professedly, 2 out of 8 patients presented with gait disturbance as the initial symptom, while another 2 out of 8 exhibited signs of tremors and recurrent falls. Additionally, one patient presented with vision changes, and difficulties in language were reported in 2 out of 8 patients, either in terms of articulation or fluency and 1 out of 8 patients was brought to medical attention because family was concerned about developmental delays, which was described as an overall concern about the lack of progress in neurologic development specifically in language and fine motor domains.

It is noteworthy, that the first clinical symptoms, prompting the family to seek medical attention is parent reported and is described here as it was noted in medical records. It is important to mention that detailed clinical and neuropsychological assessments were not conducted at the time of onset to provide detailed characterization of specific nature and extent of language difficulties or developmental delays.

Another noteworthy observation emerged during retrospective analysis, that the majority of patients, 6 out of 8 to be specific, had a paucity in language development, occurring at or right after 2 years of age. This plateau in language development, however, did not attain a level of significance which would either prompt families to seek medical attention or alert the primary care physician to pursue further diagnostic testing.

##### Neurological Symptoms

###### a. Developmental Regression:

####### Gait Problems:

**Figure 1:**
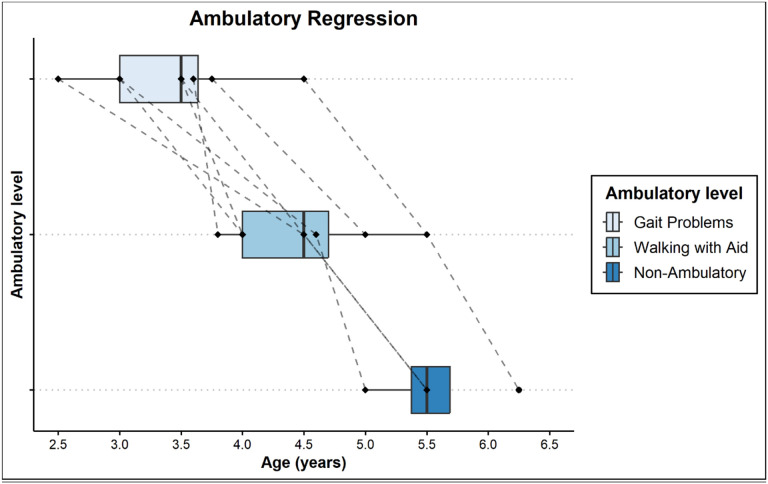
Box plots of the age of onset for stages of ambulatory regression. Figure 1 shows box plots of the age of onset for stages of ambulatory regression. Stages range from gait problems (light blue) down to non-ambulatory status (dark blue). Ages range from roughly 3 years of age to around 6 years of age. Each line corresponds to one of the 8 individual patients.

**Figure 2:**
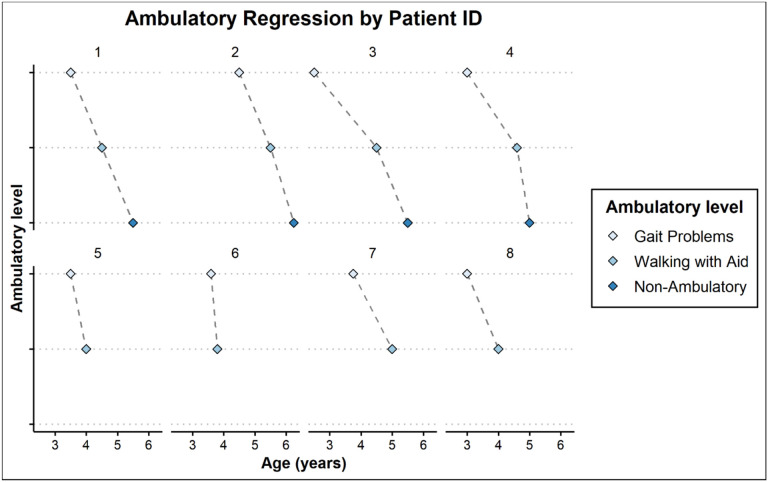
Dot plot of ambulatory regression. Figure 2 Dot plot of ambulatory regression showing distribution of the age of onset of gait problems (Gait), onset of walking with aid (Aid), and onset of being non-ambulatory (None).

Gait disturbance was reported by families as early as 2 and a half years of age and as late as 4 and half years of age. Gait progressively deteriorated requiring an aid to walk within 1–2 years from onset of gait difficulty (Table 3). Once walking difficulties were noticed, the earlier level of function was never obtained. All the patients who were seen at advanced disease stage were non-ambulatory within 1 year of age when they needed help with walking (Figures 1 and 2).

####### Language Difficulties:

Language difficulties or delays were noted as early as 2 years of age in 3/8 patients, 2.5 years in 3/8 patients, 3.5 years in 1/8 patients and by 4 years in 1/8 patients. All the patients at the time of visit had moderate to severe language issues.

###### b. Seizures:

In this patient cohort the onset of seizures ranged from 3 years and 5 months to 4 years and 10 months. Various seizure types were reported including myoclonic, atonic, and bilateral tonic-clonic seizures. In addition to seizures, myoclonic jerks were reported in all but one study participant.

In this study cohort, the majority (6 out of 8) of patients required more than one anti-seizure medication to achieve adequate seizure control. The most common anti-seizure medication used in this study cohort was Valproic acid, and 6 out of 8 patients were taking Valproic acid. In one patient, Valproic acid was stopped later in the disease course and replaced with an alternative medicine. Clobazam and Levetiracetam were the second most common medications used and 5 out of 8 patients were taking these anti-seizure medications. Lamotrigine was also used for seizure control in 2 out of 8 patients in this cohort. Cannabidiol was used in 2 out of 8 patients. One study patient was on Rufinamide and Clonazepam. There was no clear benefit of one anti-seizure medication over the other, however, this data is not sufficient to establish such a correlation. One patient had excessive sedation on combination of Clobazam and Cannabidiol, however there were no other harmful effects or side effects reported for any other anti-seizure medication at the time of the study. It should be noted that one of the limitations of the retrospective study is paucity of data and a complete profile of longitudinal use of anti-seizure medications along with the side effects and interactions of various medications is not available to report in some cases.

#### Neuro-imaging findings:

Abnormal MRI results were observed in all (8/8) cases reviewed. All patients had generalized findings with diffuse cortical and cerebellar atrophy with white matter involvement (see Table 4). Diffuse white matter involvement with T2 hyperintensities were noted in 7/8 cases and periventricular gliosis was reported in 2/8 cases. Thalami were involved in all (8/8) cases with a variable degree of volume loss and gliosis.

#### Electrophysiological findings

**Figure 3:**
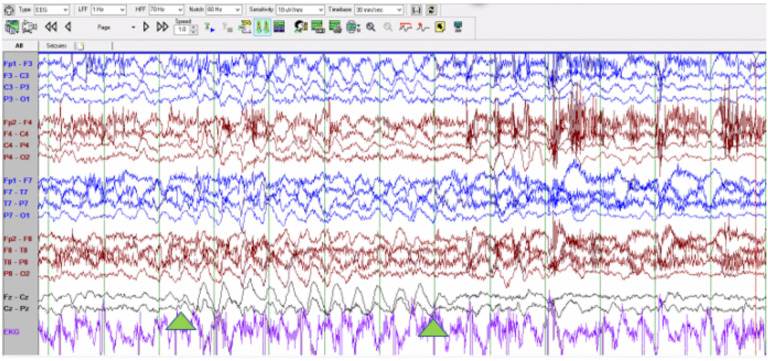
Interictal background showing generalized rhythmic delta activity. Figure 3 Interictal background showing generalized rhythmic delta activity (between green arrows).

**Figure 4:**
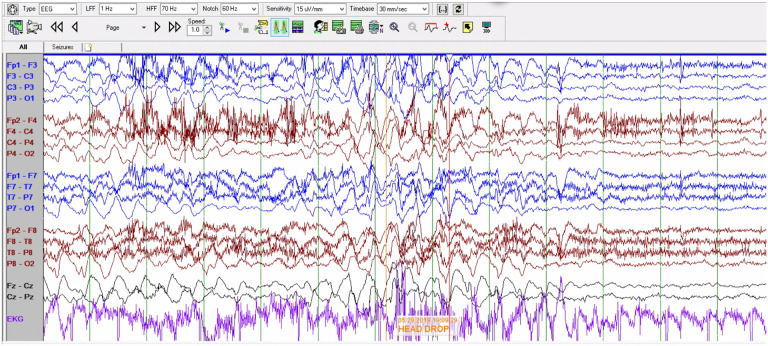
Electro-clinical seizure activity. Figure 4 Electro-clinical seizure clinically characterized by head drop with a generalized spike and wave ictal correlation.

Electroencephalographic interictal background activity for all eight patients was abnormal. Interictal background activity showed continuous generalized delta and theta slowing in 37.5% and 62.5% of patients, respectively. In 5 out of 8 patients generalized rhythmic delta activity was seen which often was maximal in the bioccipital or bifrontal regions (Figure 3). Independent multifocal spikes (100% of patients) along with generalized (75% of patients) with posterior maximum epileptiform discharges were seen. During photic stimulation no electroretinogram artifact, photomyoclonus or photoparoxysmal responses were seen in 6 of 8 patients. In one patient photic stimulation triggered a focal motor (myoclonic) seizure with concomitant spike and wave activity seen in the bioccipital regions. One unprovoked seizure was captured, identified by the caregiver, and characterized clinically by a brief head drop (atonic seizure). Ictal EEG showed a generalized spike and wave correlate (Figure 4). One event of non-epileptic staring was seen with no electrographic correlation.

#### Neuropsychological assessment results

**Figure 5:**
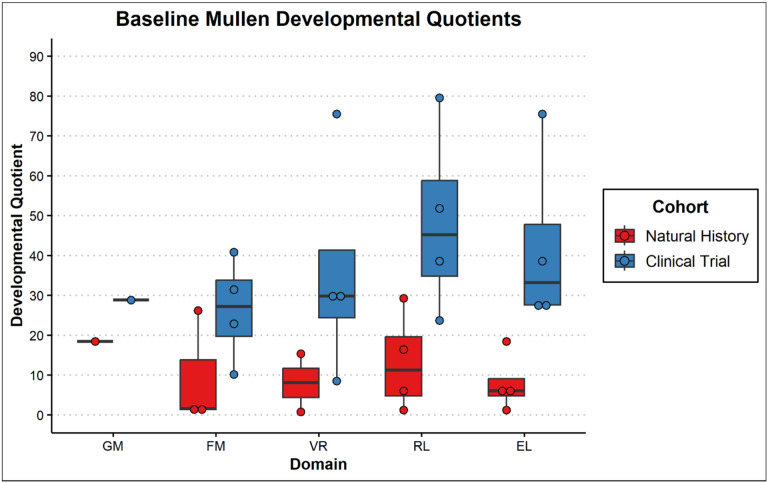
Boxplots of Developmental Quotients from the Mullen for subjects 1–8. Figure 5 Boxplots of Developmental Quotients from the Mullen for subjects 1–8. The scores represented are the Gross Motor (GM), Fine Motor (FM), Visual Reception (VR), Receptive Language (RL), and Expressive Language (EL) subtests. Subjects from the natural history study (NH) are colored red, and subjects from the gene therapy clinical trials (Clinical) are colored blue.

**Figure 6:**
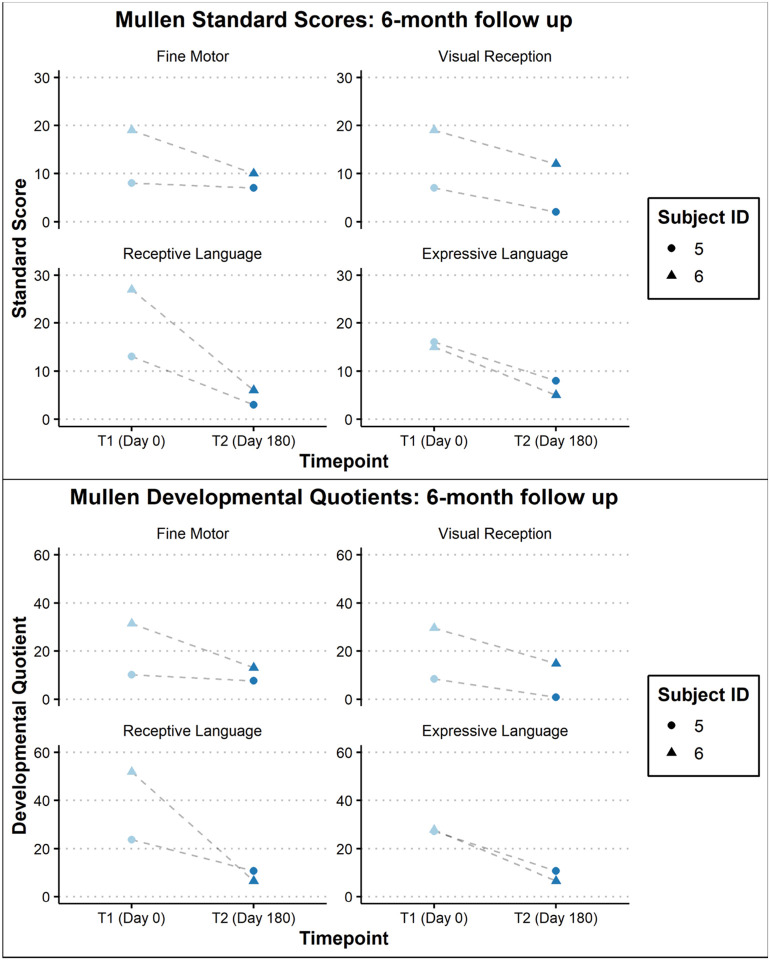
Dot plot of the Mullen Standard Scores and Developmental Quotients for 2 subjects. Figure 6 Dot plot of the Mullen Standard Scores and Developmental Quotients for 2 clinical trial subjects from the baseline data (T1) and after the course of 1 year (T2).

Assessment of developmental/cognitive abilities was limited for this population given advanced disease progression. Of the 8 children seen, 6 had complete Mullen administrations (participants 3–8). The other two were both evaluated on receptive and expressive language and only one (participant 1) on fine motor abilities. Except for participant 8, all others obtained a standard score at the floor (i.e., lowest score available) of the measure (Standard Score = 49) and participant 8 performed in the exceptionally low range (Early Learning Composite Standard Score = 63). Additionally, participants 1, 2, and 7 were outside of the normative age-range. As such, Developmental Quotients are thought to best capture the participants’ functioning and are presented in Figure 5. Participants 5 and 6 underwent follow-up evaluations with the Mullen demonstrating regression in all domains for both participants (Figure 6). The slope of the regression was most significant in the area of receptive language.

**Figure 7:**
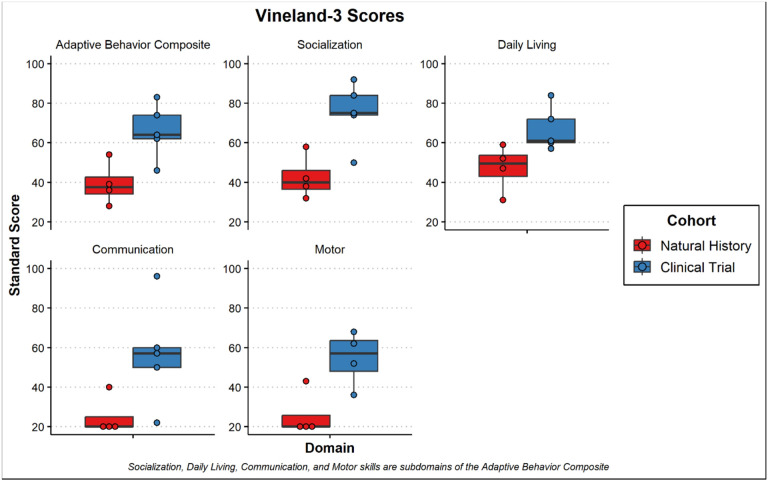
Boxplots of Standard Scores from the Vineland-3 for subjects 1–8. Figure 7 Boxplots of Standard Scores from the Vineland-3 for subjects 1–8. The scores collected are the Adaptive Behavior Composite made up of the other subdomains including Socialization, Daily Living Skills, Communication, and Motor Skills composites. Subjects from the natural history study (NH) are colored red, and subjects from the gene therapy clinical trials (Clinical) are colored blue.

**Figure 8:**
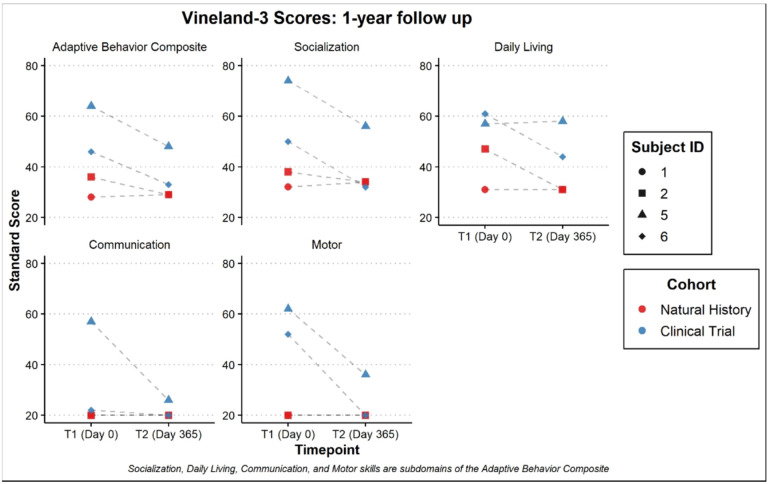
Dot plot of the Vineland-3 composite and domain scores baseline to 1 year follow up. Figure 8 Dot plot of the Vineland-3 composite and domain scores for 2 natural history subjects (ID: 1 and 2) and 2 clinical trial subjects (ID: 5 and 6) from the baseline data (T1) and after the course of 1 year (T2). Scores represented are the Adaptive Behavior Composite made up of the other subdomains including Socialization, Daily Living Skills, Communication, and Motor Skills composites.

**Figure 9:**
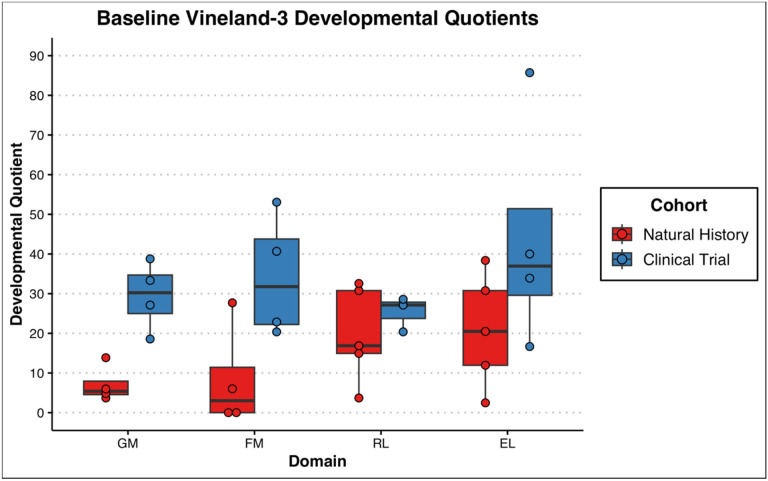
Boxplots of Developmental Quotients Scores from the Vineland-3 for subjects 1–8. Figure 9 Boxplots of Developmental Quotients Scores from the Vineland-3 for subjects 1–8. The scores represented are the Gross Motor (GM), Fine Motor (FM), Receptive Language (RL) and Expressive Language (EL) subscales. Subjects from the natural history study (NH) are colored red, and subjects from the gene therapy clinical trials (Clinical) are colored blue.

With respect to adaptive functioning, there was noted to be significant variability although all but participant 8 was noted to be in the exceptionally low range on the Vineland-3 Adaptive Behavior Composite (Standard Score mean = 51.5 ± 31.5). Composite standard scores for the adaptive measure are presented in Figure 7. Participants in the clinical trial were rated overall at a higher level of functioning than the participants in the natural history portion. Similar to results from the Mullen, follow-up evaluations on the Vineland-3 for participants 1, 2, 5 and 6 also demonstrated parent-reported regression overall (Adaptive Behavior Composite; see Figure 8). Individually, participant 5 had sharp regressions in Communication while the others remained at the floor of the subdomain. Daily Living remained stable for participants 1 and 5 but was noted to regress for participants 2 and 6. Social skills were also relatively stable for participants 1 and 2 but showed a decline in 5 and 6. Further, Motor Skills were already at the floor for participants 1 and 2 but demonstrated rapid regression for participants 5 and 6. A look at the Developmental Quotients for the motor and communication subscales of the Vineland-3 revealed smaller differences between natural history and clinical trial participants. Further, the language scales were rated more highly than motor scales (Figure 9).

**Figure 10:**
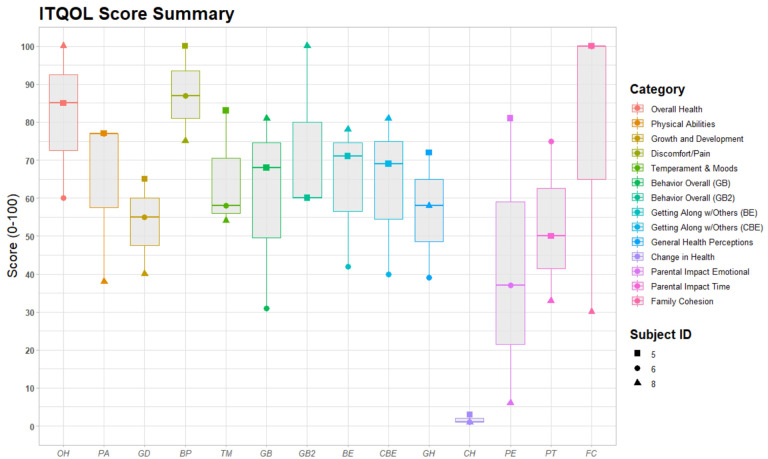
Boxplots of the ITQOL scores for 3 clinical trial subjects. Figure 10 Boxplots of the ITQOL scores for 3 clinical trial subjects. Scores represented are Overall Health (OH), Physical Abilities (PA), Growth and Development (GD), Discomfort/Pain (BP), Temperament and Moods (TM), Behavior Overall (GB & GB2), Getting Along with Others (BE & CBE), General Health Perceptions (GH), Change in Health (CH), Parental Impact Emotional (PE), Parental Impact Time (PT), and Family Cohesion (FC).

**Figure 11:**
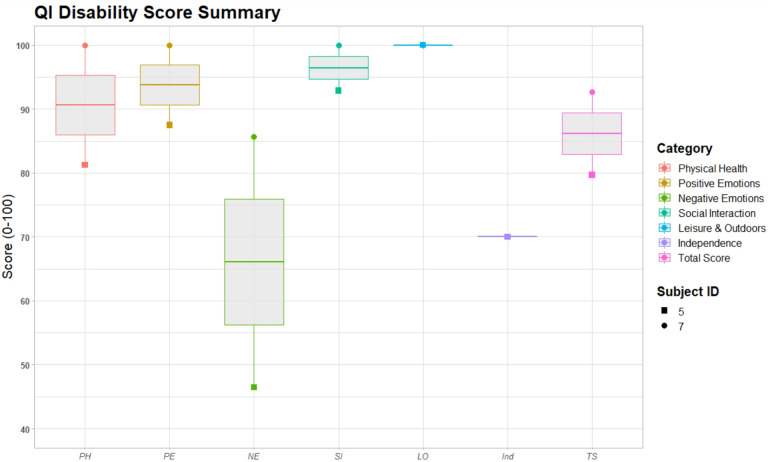
Boxplots of the QI-Disability scores for 2 clinical trial subjects. Figure 11 Boxplots of the QI-Disability scores for 2 clinical trial subjects. Scores represented are Physical Health (PH), Positive Emotions (PE), Negative Emotions (NE), Social Interaction (SI), Leisure & Outdoors (LO), Independence (Ind), and Total Score (TS).

For the clinical trial portion, assessment of health-related quality of life was assessed (participants 5–8). Three participants received the ITQOL (participants 5, 6, and 8). There was significant variability in responses (Figure 10), but parents rated Overall Health as good to excellent. While parents for participants 6 and 8 rated their child’s Change in Health much worse than one year ago, parents of participant 5 rated it to be about the same. Parental Emotional Impact and Family Cohesion also revealed significant variability. The QI-Disability was administered to participants 5 and 7 (Figure 11). The greatest variability was seen in Negative Emotions (e.g., behavioral outbursts, withdrawn behavior), but both families rated generally positive Physical Health, Positive Emotions (e.g., smiling, laughing), Social Interaction, and Leisure Skills.

## Discussion

Clinical reports and research in CLN7 have documented neurodevelopmental regression in cognitive, language and motor abilities along with seizures.^12,13^ Our comprehensive cross-sectional and retrospective review outlines valuable information integrating the clinical findings with standardized neuropsychological assessments, electrophysiologic and neuroimaging findings. Although our sample size is small, it shows a geographically and culturally diverse cohort of patients.

In this study, median age of onset is 3.4 years with median age of diagnosis at 4.8 years. It is also important to note that onset of symptoms was noted no later than 4 years of age in all patients (8/8). Patients had a median diagnostic delay of 18 months from symptom onset to diagnosis, which suggests a delay in recognition by clinicians. This delay in diagnosis of CLN7 is due to the rarity of CLN7 deficiency, paucity of clinical data reported in current literature and lack of awareness. With this study data, we aim to add to descriptive phenotype of CLN7 disease through standardized neuropsychological and quality of life assessment tools.

Common first symptom reported by the family which led to seek medical attention was gait disturbance in 2/8 patients, “shaking” and falling in 2/8 patients, language difficulty or delay in 2/8 patients. it is also important to note that paucity in language development was reported in 6/8 patients. This plateau in language was noted right after the age of 2 years, however not significant enough to seek medical attention.

In the present study all patients developed seizures, followed by relentless neurologic regression with loss of motor and language functions. Electroencephalogram (EEG) findings in variant late infantile neuronal ceroid lipofuscinosis type 7 (CLN7) have been poorly described in the literature. In our case study of eight patients, video-EEG findings were non-specific and consistent with a multifocal and/or generalized epileptic encephalopathy with an elevated risk for seizures. Two epileptic seizures were captured in two different patients and were identified as a myoclonic and an atonic seizure. Ictal EEG showed a bi-occipital non-lateralizing onset triggered by photic stimulation and a generalized ictal onset, respectively. Further studies are needed to reach a more conclusive description of electro-clinical patterns of patients with CLN7.

Analysis of the CLN7 mutations in our patient population revealed 12 distinct pathogenic variants, out of which 6 variants are novel to this study and have not been described in literature before.

Abnormal MRI results were observed in all (8/8) cases reviewed. All patients had generalized findings with diffuse cortical and cerebellar atrophy with white matter involvement. Diffuse white matter involvement with T2 hyperintensities were noted in 7/8 cases and periventricular gliosis was reported in 2/8 cases. Thalami were involved in all (8/8) cases with a variable degree of volume loss and gliosis. Cognitively and adaptively, participants had declines in functioning seen both when compared to peers as well as longitudinally for those with follow-up data. Participants in the natural history portion also performed more poorly than those in the clinical trial thought to be secondary to longer effects of the disease.

The study limitations include retrospective nature and small sample size and missing data. Another limitation is lack of long term follow up of these patients. Further, due to decline in patients, there are very limited tools to assess their neurodevelopmental abilities. While the use of raw scores and developmental quotients helped to be able to better capture abilities, there are no current tests that can fully capture the lower end of the disease spectrum. There are also linguistic and cultural considerations for such a rare disease that are difficult to overcome in a singular study. Our considerations for this are further detailed in Bordes Edgar et al.^11^

## Conclusions

This study provides a comprehensive description of CLN7 disease, using medical history questionnaires and standardized tools. There is description of more than 121 patients in literature (Table 1) based upon retrospective chart reviews. Our study adds data to elaborate on clinical phenotype, neuroimaging and electrophysiologic findings along with standardized neuropsychological assessments. We aim to explore the role of standardized testing that can not only describe the depth and breadth of disease phenotype but can also be used as clinical endpoint points in a future clinical trial. We believe the data from this study would be instrumental with future therapy trials as it provides a baseline for assessment of improvement. A prospective natural history study would provide even more useful information for clinical and further research and would be the next step for future studies.

## Figures and Tables

**Table 1:** Known Mutations Table

Mutation, nucleotide change^11^	Mutation, protein change	Number of Patients with Mutation	Patient Location	Citations	Citation Number in List
**c.863+3_4insT**	Altered Splicing	3	Italy	Aiello *et al.,* 2009	5
**c.929G>A**	p.Gly310Asp	4	Turkey; Italy; France	Topgu *et al.,* 2004; Siintola *et al.,* 2007; Aiello *et al.,* 2009; Poncet *et al.,* 2022	14; 1; 5; 15
**c.627_643del17**	p.Met209IlefsX3	1	Italy	Aiello *et al.,* 2009	5
**c.154G>A**	p.Gly52Arg	1	Italy	Aiello *et al.,* 2009	5
**c.1444C>T**	p.Arg482*	4	France; China; Scandinavian; United States	Aiello *et al.,* 2009; Ren, 2019; This Study	5; 16
**c.1141G>T**	p.Glu381*	7	France; Netherlands	Aiello *et al.,* 2009; Roosing *et al.,* 2015; Poncet *et al.,* 2022	5; 4; 15
**c.863+1G>C**	Altered Splicing	2	Italy; Turkey	Aiello *et al.,* 2009; Kousi *et al.,* 2009	5; 2
**c.2T>C**	p.Met1Thr	1	Italy	Aiello *et al.,* 2009	5
**c.1340C>T**	p.Pro447Leu	1	Italy	Aiello *et al.,* 2009	5
**c.881C>A**	p.Thr294Lys	27	Italy; Turkey; Czech Republic; Romania; Egypt; Hungary; France	Aiello et al., 2009; Kousi et al., 2009; Craiu et al., 2015	5; 2; 17; 18; 15; 19
				Refeat et al., 2022; Poncet *et al,* 2022; Jilani, *et al.,* 2019; This Study	
**C.103C>T**	p.Arg35*	6	Italy; Turkey; Argentina; England; Paraguay	Aiello *et al.,* 2009; Kousi *et al.,* 2009; Kohan *et al,* 2015; Khan *et al,* 2017; This Study	5; 2; 20; 21
**C.1398C>T**	p.Pro412Leu	3	Saudi Arabia	Aldahmesh *et al.,* 2009	12
**c.416G>A**	p.Arg139His	4	India; Egypt	Kousi *et al.,* 2009; Refeat *et al.,* 2022; Jilani, *et al,* 2019	2; 18; 19
**c.468_469delinsCC**	p.Thr156_Ala157delins, Thr156_Pro157	1	Netherlands	Kousi *et al,* 2009	2
**c.627_643del**	p.Met209Ilefs*3	2	Italy	Aiello *et al.,* 2009; Kousi *et al.,* 2009	5; 2
**c.1103-2delA**	Altered Splicing	1	Czech Republic	Kousi *et al.,* 2009	2
**c.1393C>T**	p.Arg465Trp	1	Albania/Greece	Kousi *et al.,* 2009	2
**c.259C>T**	p.Gln87*	1	Canada	Kousi *et al.,* 2012	3
**c.479C>A**	p.Thr160Asn	1	Turkey	Kousi *et al.,* 2012	3
**c.479C>T**	p.Thr160Ile	2	Cook Islands; Egypt	Kousi *et al.,* 2012; Refeat *et al,* 2022	3; 18
**c.554-1G>C**	Altered Splicing	1	Romania	Kousi *et al.,* 2012	3
**c.754+1G>A**	Altered Splicing	2	Turkey	Kousi *et al.,* 2012	3
**c.1373C>A**	p.Thr458Lys	2	Romania	Kousi *et al.,* 2012; This Study	3
**c.1394G>A**	p.Arg465Gln	5	Turkey; England	Kousi *et al.,* 2012; Khan *et al,* 2017; Jilani, *et al,* 2019	3; 21; 19
**c.1408A>G**	p.Met470Val	1	Turkey	Kousi *et al.,* 2012	3
**c.1420C>T**	p.Gln474*	1	Turkey	Kousi *et al.,* 2012	3
**c.472G>A**	p.Gly158Ser	5	Israel	Mandel *etal.,* 2014	22
**whole gene deletion**	whole gene deletion	1	China	Qiao *et al.,* 2022	23
**c.136_137delAT**	p.Met46fs	1	China	Qiao *et al,* 2022	23
**c.750A>G**	Altered Splicing	4	Turkey; France	Reith *et al.,* 2022; Poncet *et al.,* 2022	24; 15
**c.697A>G**	p.Arg233Gly	1	Turkey	Topgu *et al.,* 2004; Siintola *et al.,* 2007	14; 1
**c.754+2T>A**	Altered Splicing	14	Turkey; Czech Republic; Romania; Hungary	Topgu *et al.,* 2004; Siintola *et al.,* 2007; Kousi *et al.,* 2009; Craiu *et al.,* 2015; Jilani, *et al.,* 2019; This Study	14; 1; 2; 17; 19
**c.894T>G**	p.Tyr298*	1	India	Siintola *et al.,* 2007	1
**c.1102G>C**	p.Asp368His	3	Turkey; Netherlands; United States	Siintola *et al.,* 2007; Roosing *et al.,* 2015; Kim *et al.,* 2019	1; 4; 25
**SVA insertion**	Altered Splicing	1	United States	Kim *et al.,* 2019	25
**c.1286G>A**	p.Gly429Asp	1	Turkey	Topgu *et al.,* 2004; Siintola *et al.,* 2007	14; 1
**c.362A>G**	p.Tyr121Cys	3	Egypt	Stogmann *et al.,* 2009	13
**c.1361T>C**	p.Met454Thr	14	India; Turkey; Iran	Patino *et al.,* 2014; Khan *et al.,* 2017; Zare-Abdollahi *et al.,* 2019	26; 21; 32
**c.1219T>C**	p.Trp407Arg	3	India	Patino *et al.,* 2014	26
**c.1006G>C**	p.Glu336Gln	15	Netherlands; England; France	Roosing *et al.,* 2015; Khan *et al.,* 2017; Poncet *et al.,* 2022	4; 21; 15
**c.233G>A**	p.Trp78*	2	England	Khan *et al.,* 2017	21
**c.554-5A>G**	Altered Splicing	1	China	Ren *et al.,* 2019	16
**c.525T>A**	p.Cys175*	1	Russia	Kozina *et al.,* 2018	27
**c.325_339del**	p.Val109_Ile113del	1	Iran	Bereshneh & Garshasbi, 2018	28
**c.439+3A>C**	p.Ile67Glufs*3	1	not reported	Bauwens *et al.,* 2019	29
**c.590del**	p.Gly197Valfs*2	1	not reported	Bauwens *et al.,* 2019	29
**c.721G>T**	p.Gly241*	1	Turkey	Kose *et al.,* 2021	30
**c.1445G>C**	p.Arg482Pro	1	Germany	Birtel *et al.,* 2018	31
**c.1235C>T**	p.Pro412Leu	8	Iran; Egypt	Zare-Abdollahi *et al.,* 2019 ; Refeat *et al.,* 2022	32; 18
**c.1093C>T**	p.Gln365*	2	Turkey	Kose *et al.,* 2021	30
**c.1391C>T**	p.Ala464Val	1	Poland	Ziora-Jakutowicz *et al.,* 2019	33
**c.301G>C**	p.Ala146Pro	1	Turkey	Kose *et al.,* 2021	30
**c.63-1G>A**	Altered Splicing	2	India; not reported	Gowda *et al.,* 2020; Dozieres-Puyravel et al., 2020	34; 35
**c.886G>C**	p.Asp269His	1	Egypt	Refeat *et al.,* 2022	18
**c.600G>A**	p.Trp200Ser	1	Egypt	Refeat *et al.,* 2022	18
**c.863 + 2dup (G>A)**	Altered Splicing	1	Italy	Pasquetti *et al.,* 2023	36
**c.850G>C**	p.Ala284Pro	1	Bangladesh	Rahman *et al.,* 2021	37
**c.1241_1242insGAAT**	p.Ile414Metfs*109	1	Not reported	Jilani *et al.,* 2019	19
**c.863+4A>G**	not reported	1	Not reported	Jilani *et al.,* 2019	19
**c.1351-G>A**	p.Phe186Cys	1	China	Niu *et al,* 2022	38
**c.557T>G**	p.Phe186Cys	1	China	Niu *et al.,* 2022	38
**c.755-2726_998+1981delinsGTA**	p. Ser253Leufs*79	1	France	Poncet *et al.,* 2022	15
**c.104G>A**	p.Arg35Gln	1	France	Poncet *et al.,* 2022	15
**c.155G>C**	p.Gly52Ala	1	France	Poncet *et al.,* 2022	15
**c.1265C>A**	p.Ser422*	1	France	Poncet *et al.,* 2022	15
**c.1009C>T**	p.Arg337Cys	1	France	Poncet *et al.,* 2022	15
**c.998+1669A>G**	p.Lys333Asnfs*18	1	France	Poncet *et al,* 2022	15
**c.1351-2A>G**	Altered Splicing	1	India	This Study	N/A
**c.1036delG**	p.Val346LeufsTer68	1	United States	This Study	N/A
**c.440-2A>T**	Altered Splicing	1	United States	This Study	N/A
**c.198+2T>C**	Altered Splicing	1	Saudi Arabia	This Study	N/A
**c.1206del**	p.Ile403LeufsTer11	1	United States	This Study	N/A
**c.1437G>A**	p.Trp479*	1	Sweden	This Study	N/A

**Table 2:** Demographics- participants gender, country of origin and genotype

Subject ID	Gender	Country/Language	Affected Gene	Mutation Information	Zygosity	Predicted DNA Variation
1	Female	India-USA/English	MFSD8	c.1351-2A>G	Homozygous	Splice acceptor
2	Male	USA/English	MFSD8	c.1036delG (p. Val346LeufsTer68) c.440-2A>T	Compound Heterozygous	Frameshift Splice acceptor
3	Female	Sweden/Swedish	MFSD8	c.1437G>A, (p.Trp479Ter) c.1444C>T (p.Arg482Ter)	Compound Heterozygous	Stop gain Stop gain
4	Male	Hungary/Hungarian	MFSD8	c.881C>A, p.(Thr294Lys) c.754+2T>A	Compound Heterozygous	Missense Splice donor
5	Female	Paraguay/Spanish	MFSD8	c.103C>T, (p.Arg35Ter)	Homozygous	Stop gain
6	Male	Romania/Romanian	MFSD8	c.1373C>A, (p.Thr458Lys)	Homozygous	Missense
7	Female	Saudi Arabia/Arabic	MFSD8	c.198+2T>C	Homozygous	GT Splice donor
8	Female	USA/English	MFSD8	C.1444C>T (p. Arg482Ter) C.1206del (p.Ile403LeufsTer11)	Compound Heterozygous	Stop gain Frameshift

**Table 3:** Developmental regression

Subject ID	Age at Visit (yy.mm)	Age at Onset of first symptom (yy.mm)	Age at Diagnosis (yy.mm)	Initial Symptom	Seizure onset (yy.mm)	Onset of Gait Problems (yy.mm)	Onset of Language difficulties (yy.mm)	Onset of Cognitive issues (yy.mm)	Onset of Vision Changes (yy.mm)
1	6.9	3.4	4.4	Gait Problems	4	3.5	4	3.5	5
2	6.11	4	4.75	Vision change	4.4	4.5	2.5	Unsure	4
3	5.5	2.4	5	Gait problems	4.8	2.5	2.5	3.5	2.5
4	5.6	3	5	DD	3.5	Unsure	2	4.5	No vision problems
5	5	2	4.4	Language issue	3.5	3.5	2	3.5	Undetermined
6	4.7	3.8	4	Shaking/fall	3.8	Intermittent with myoclonus at onset of symptoms	3.5	Unsure	Undetermined
7	5.11	3.75	5.25	Shaking/falls	4	4.5	2	Unsure	Undetermined
8	4.1	3	4	Paucity of language and motor development	3.5	2.5	2.5	2–3	Undetermined

**Table 4:** Neuro-imaging findings

Subject ID	Age (years. months)	MRI Brain
1	6.9	Significant progressive cerebral and cerebellar atrophy. Faint regions of T2 signal hyperintensity in the periventricular white matter Bilateral thalamic atrophy
2	6.11	Generalized parenchymal volume loss and periventricular gliosis. White matter pallor on T2 Flair images with increased signal intensity in periventricular white matter and posterior limbs of internal capsule Thalamic atrophy and gliosis
3	5.5	Diffuse supratentorial and infratentorial atrophy White matter appropriately myelinated Mild thalamic volume loss with gliosis
4	5.6	Generalized parenchymal volume loss Periventricular gliosis Paucity of white matter Increased T2 signal abnormalities along periventricular white matter Thalamic volume loss
5	5	Mild to moderate generalized cerebral and cerebellar atrophy associated Abnormal myelin pallor involving the deep cerebral white matter and corticospinal tracts Marked thalamic atrophy
6	4.7	Generalized mild cerebral and moderate cerebellar atrophy. Confluent myelin pallor involving the deep cerebral white matter and extending along the corticospinal tracts. Moderate thalamic atrophy
7	5.11	Mild to moderate generalized cerebral and cerebellar atrophy. Moderate ventriculomegaly White matter paucity Mild myelin pallor in the periventricular white matter
8	4.1	Mild to moderate generalized cerebral atrophy with nonobstructive ventricular enlargement Symmetric myelin pallor involving the deep cerebral white matter with moderate bilateral thalamic atrophy

## Data Availability

The data was extracted from the participants electronic medical records as well as paper source data from participation in the natural history study and clinical trial. The datasets generated during and/or analyzed during the current study are not publicly available due to protection of participants privacy and protected health information. A password protected; de-identified data set stored on the secure servers at Childrens Health can be available from the corresponding author on reasonable request.
